# SG-APSIC1056: Finding the right fit: Our experience in quantitative N95 respirator fit-testing

**DOI:** 10.1017/ash.2023.61

**Published:** 2023-03-16

**Authors:** Jin Min Sheena Ong, Bushra Binte Shaik Ismail, Sheena Ong Jin Min, Gillian Lee Li Xin, Lee Lai Chee, Molly How Kue Bien, Ling Moi Lin

**Affiliations:** Singapore General Hospital, Singapore; Singapore General Hospital, Singapore; Singapore General Hospital, Singapore; Singapore General Hospital, Singapore; Singapore General Hospital, Singapore; Singapore General Hospital, Singapore; Singapore General Hospital, Singapore

## Abstract

**Objectives:** Following a cluster of COVID-19 cases in a Singapore public hospital in April 2021, the local health authority mandated the use of N95 respirators in all inpatient wards. This increased the demand for N95 mask fit-testing to ensure that healthcare workers were donning respirators that fit their facial characteristics and hence provided protection through a good facial seal. The demand for fit-testing during the pandemic highlighted the scarcity of manpower and ergonomics concern, such as carpel tunnel syndrome experienced in long hours of qualitative fit-testing sessions. We evaluated the operational efficiency, cost-effectiveness, and difference in passing rate after the introduction of the quantitative method. **Methods:** Conventional qualitative fit-testing was conducted using manual pumping of a challenge agent, enabling the user to determine the fit of the respirator. The quantitative fit-testing protocol used a condensation particle counter (CPC) to measure the concentration of particles inside the mask and the atmosphere to determine the fit of respirator. The Occupational Safety and Health Administration (OSHA)–approved minimum fit factor of 100 was used as the criterion for a successful N95 respirator fit. Tubes used during quantitative fit-testing were reprocessed using thermal disinfection. **Results:** Quantitative mask fit-testing provided an objective numerical measure to assess adequate fit of N95 respirator, which provided users with confidence in the respirator fit. It addressed a manpower limitation issue because it did not require qualified trainers to conduct the test, and automation also prevented any potential occupational hazard from repeated actions required in qualitative fit-testing. An increase in the passing rate for N95 fit-testing from 94.5% to 95.5% was observed. However, the high cost of equipment, annual recalibration, and consumables must be considered. **Conclusions:** Quantitative N95 fit-testing, when adopted with careful consideration of its cost, is an approach to consider for hospital-wide fit-testing.

